# Magnetic field role in solar-driven water evaporation of Fe_3_O_4_/polyvinyl alcohol

**DOI:** 10.1039/d5ra07635h

**Published:** 2025-12-15

**Authors:** Nguyen Thi Bich Ngoc, Nguyen Minh Hoang, Vu Thi Trang, Le Tuan Tu, Ta Quynh Mai, Hung-Anh Tran Vu, Nang Xuan Ho, Hong-Ha Thi Vu, Vu Anh Doan, Phuong Anh Lam, Van-Duong Dao

**Affiliations:** a Faculty of Biotechnology, Chemistry and Environmental Engineering, PHENIKAA School of Engineering, Phenikaa University Hanoi 12116 Vietnam duong.daovan@phenikaa-uni.edu.vn; b Phenikaa University Nano Institute, Phenikaa University, PHENIKAA School of Engineering Hanoi 12116 Vietnam; c Faculty of Physics & Biophysics, Vietnam Medical Military University 160 Phung Hung Hanoi 10000 Vietnam; d Faculty of Physics, University of Science, Vietnam National University, Hanoi 334 Nguyen Trai Hanoi 10000 Vietnam; e Faculty of Vehicle and Energy Engineering, Phenikaa School of Engineering, Phenikaa University Hanoi 12116 Vietnam nang.hoxuan@phenikaa-uni.edu.vn; f School of Material Science, Hanoi University of Science and Technology Hanoi Vietnam; g Department of Chemistry, Case Western Reserve University 10900 Euclid Avenue Cleveland Ohio 44106 USA

## Abstract

Magnetically responsive materials offer unique opportunities for enhancing solar steam generation (SSG) through externally induced structural reconfiguration. In this study, we investigate the influence of magnetic field strength on the photothermal behavior of Fe_3_O_4_/polyvinyl alcohol (PVA)-based membranes. Under a moderate magnetic field (1876 G), the membrane self-organizes into a stable three-dimensional needle-like architecture that promotes broadband solar absorption, efficient thermal localization, and a high evaporation rate of 1.3236 kg m^−2^ h^−1^ under 1 sun illumination with the solar efficiency of 93.30%. However, when exposed to stronger magnetic fields (≥2355.9 G), the microstructure becomes distorted and destabilized, resulting in diminished light-harvesting capability and suppressed evaporation performance. These findings highlight the delicate balance between structural enhancement and degradation in magnetic field-assisted photothermal systems and demonstrate the critical role of controlled field strength in optimizing the efficiency of SSG devices.

## Abstract 2

Magnetically responsive materials offer unique opportunities for enhancing solar steam generation (SSG) through externally induced structural reconfiguration.

## Graphical abstract

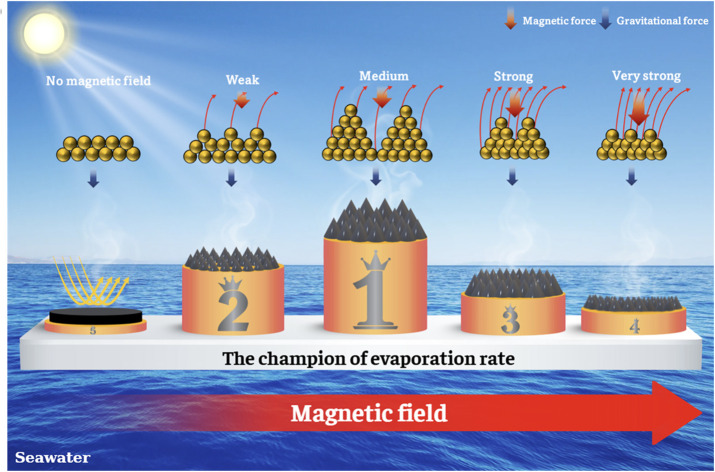

## 1. Introduction

The global demand for clean and safe freshwater is escalating, driven by population growth, industrialization, and the increasingly adverse impacts of climate change. Conventional desalination technologies, such as reverse osmosis, multi-stage flash distillation, and vapor compression, are widely deployed for large-scale water purification.^1,2^ However, their dependence on high-pressure systems, thermal energy, and complex maintenance procedures makes them economically and logistically infeasible for remote or under-resourced regions.^3–5^ In contrast, solar steam generation (SSG), which utilizes abundant solar energy to drive interfacial evaporation, has emerged as a promising and sustainable alternative for decentralized water treatment.^6,7^

The performance of SSG devices is inherently linked to the properties of the photothermal absorber, which must efficiently convert solar energy into localized heat, facilitate rapid water transport to the evaporation interface, and enable continuous vapor release.^8–10^ To enhance solar absorption and evaporation efficiency, researchers have transitioned from traditional 1D and 2D absorber designs to advanced 3D architectures featuring hierarchical porosity and light-trapping geometries.^11–13^ These 3D configurations increase the active surface area, suppress optical losses due to reflection, and improve thermal confinement. However, most reported 3D absorbers are fabricated using fixed templates, such as 3D printing^14,15^ and biogenic scaffolds,^16,17^ resulting in static geometries that are prone to salt accumulation and fouling during prolonged operation. The inability to dynamically reconfigure these structures limits long-term performance and reduces the potential for reuse.

To overcome the limitations of conventional photothermal materials, magnetically responsive systems have garnered growing interest due to their ability to form and dynamically reconfigure spatial architectures in response to external magnetic fields.^18,19^ Among these materials, Fe_3_O_4_ nanoparticles are particularly notable because of their high saturation magnetization, low coercivity, and good compatibility.^20^ When exposed to a magnetic field, Fe_3_O_4_ nanoparticles can self-assemble into vertically aligned or needle-like 3D structures. These formations significantly enhance solar light absorption and interfacial heat localization, which are essential for efficient photothermal applications.^21,22^

The magnetic responsiveness of Fe_3_O_4_ allows real-time control over the material's geometry and enables its recyclability and structural regeneration.^23^ These features are highly beneficial for the development of durable and sustainable solar evaporation systems. However, Fe_3_O_4_ nanoparticles alone still face certain drawbacks. Their weak absorption in the visible spectrum, low surface hydrophilicity, and susceptibility to oxidation can limit their photothermal performance in practical environments.

To overcome these shortcomings, researchers have developed hybrid materials that integrate Fe_3_O_4_ with functional components such as carbon-based nanomaterials, metallic nanoparticles, or polymeric matrices.^23–26^ In these composite systems, Fe_3_O_4_ serves as the magnetic core, allowing for the formation of complex 3D architectures that facilitate water transport and localized heat retention. The additional components contribute to improved photothermal conversion efficiency, broader light absorption, and enhanced hydrophilicity.^27^ These combined effects significantly improve the overall performance and durability of solar-driven water evaporation systems, making Fe_3_O_4_-based hybrids more viable for real-world applications.

For example, Ngoc *et al.*^25^ developed a hybrid nanoparticle system by combining Fe_3_O_4_ nanoparticles with carbon dots (CDs), resulting in Fe_3_O_4_/CDs nanocomposites. Carbon dots are intrinsically hydrophilic and possess strong broadband light absorption properties. These characteristics improved water affinity at the evaporation surface and enhanced photothermal efficiency. Additionally, under the influence of a magnetic field, the Fe_3_O_4_ nanoparticles aligned to form a structured 3D network that promoted efficient water transport and localized heat concentration. As a result, the Fe_3_O_4_/CDs hybrid system achieved a significantly higher evaporation rate of 1.352 kg m^−2^ h^−1^, which is a substantial improvement compared to Fe_3_O_4_ nanoparticles alone. In another study, Zhang *et al.*^21^ developed a magnetically assisted spray-coating technique to fabricate a vertically aligned 3D tower-like Fe_3_O_4_ array on PET fabric, with tunable structural height and density controlled by spray parameters. The resulting 3D architecture achieved an exceptional solar absorbance of 98.6% across the full solar spectrum, significantly surpassing its 2D counterpart. When integrated with a yolk–shell-modified melamine–formaldehyde sponge, the composite Fe_3_O_4_/PET-mMF evaporator demonstrated a high-water evaporation rate of 1.59 kg m^−2^ h^−1^ under 1 sun illumination, providing a low-cost and efficient platform for solar steam generation.

Although numerous studies have explored the magnetic properties of Fe_3_O_4_ particles, to the best of our knowledge, no research has systematically investigated the correlation between magnetic field strength, the resulting 3D structural morphology formed by Fe_3_O_4_ particles, and the evaporation performance of the membrane. Therefore, in this work, we examine the influence of magnetic field strength on the structural evolution and photothermal evaporation behavior of Fe_3_O_4_/PVA-based membranes. We show that moderate magnetic fields induce the self-assembly of Fe_3_O_4_/PVA nanoparticles into vertically aligned needle-like configurations. These dynamic 3D structures enhance light absorption, thermal localization, and water transport, resulting in significantly improved evaporation performance under 1 sun irradiation. However, when the magnetic field exceeds 3000 G, the internal structure becomes unstable, leading to a collapse of the 3D morphology and a notable decrease in efficiency. This study offers new insight into the design of magnetically reconfigurable polymer–nanoparticle composites for solar steam generation, and presents a pathway toward scalable, recyclable, and high-performance water purification technologies.

## 2. Experiments

### 2.1. Synthesis methods

#### 2.1.1. Synthesis process of Fe_3_O_4_ nanoparticles

Fe_3_O_4_ nanoparticles were synthesized based on the previous report with some modifications.^28^ Briefly, 0.05 mol of ferric chloride hexahydrate (FeCl_3_·6H_2_O) and 0.05 mol of ferrous chloride tetrahydrate (FeCl_2_·4H_2_O) were dissolved in 25 mL of deionized water under continuous stirring at 500 rpm. A 25 mL aqueous solution of 4 M sodium hydroxide (NaOH) was then added dropwise to the mixture, which was maintained at 60 °C and stirred for 24 hours to facilitate the formation of Fe_3_O_4_. The resulting black precipitate was magnetically separated, washed three times with absolute ethanol to remove residual ions, and dried at 80 °C for 12 hours. The final product was designated as Fe_3_O_4_ nanoparticles and stored in absolute ethanol for subsequent use.

#### 2.1.2. Fabrication process of Fe_3_O_4_/PVA nanoparticles

The Fe_3_O_4_/PVA nanoparticles were fabricated according to the previous report^29^ with some modifications. 1 g of Fe_3_O_4_ nanoparticles was dispersed in 20 mL of a 2 wt% PVA aqueous solution. The mixture was subjected to magnetic stirring and ultrasonication for 30 minutes to ensure homogeneous dispersion. The resulting suspension was then dried at 80 °C for 48 hours to allow complete evaporation of the solvent, thereby promoting uniform adhesion of the PVA matrix onto the surface of the Fe_3_O_4_ nanoparticles.

### 2.2. Characterizations

The surface morphology of the samples was analyzed using a scanning electron microscope (SEM, HITACHI S-4800) and the crystallization of the samples was characterized by a transmission electron microscopy (TEM, JEM-1010, Japan) operated at an accelerating voltage between 40 kV to 100 kV. The crystallographic structure was examined using an X-ray diffractometer (MiniFlex 600, Rigaku, Japan) equipped with a Cu Kα radiation source (*λ* = 1.5406 Å), operated at 40 kV and 15 mA. Optical absorption properties were evaluated using UV-vis spectroscopy (UH4150 spectrophotometer) equipped with an integrating sphere, while the surface functional groups were identified through Fourier-transform infrared (FTIR) spectroscopy in reflectance mode using an Affinity-1S spectrophotometer (Shimadzu, Kyoto, Japan). Magnetic properties, including hysteresis behavior, were measured using a vibrating sample magnetometer (VSM, Lakeshore 7404) at a stable temperature of 300 K. The strength of the external magnetic field was estimated using a simulation tool available on the website (https://www.kjmagnetics.com/magnetic-field-calculator.asp), and the results are summarized in Table 1. Additionally, the surface temperature of the Fe_3_O_4_/PVA membrane under solar illumination was monitored using a Testo 868 infrared thermal imaging camera.

**Table 1:** The calculated magnetic field at different distances from the surface of a round disk magnet

Distance (cm)	Magnetic field (G)
0.5	2748.3
1	2355.9
1.5	1876
2	1429.2

## 3. Results and discussion

**Fig. 1:**
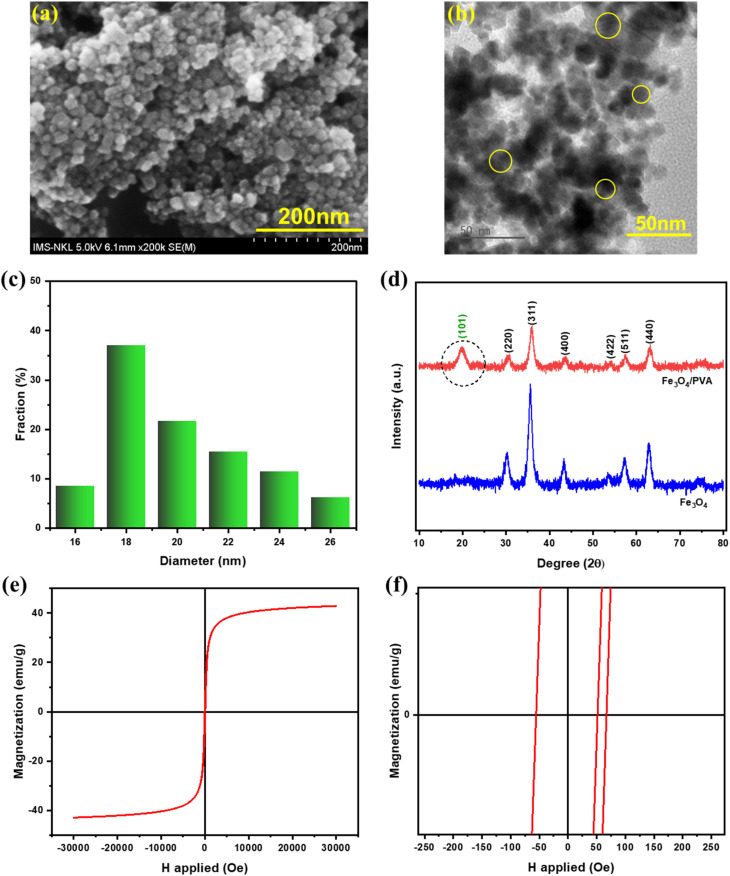
(a) SEM, (b) TEM, and (c) particle size distribution of Fe_3_O_4_/PVA calculated from the TEM image. (d) XRD pattern of Fe_3_O_4_/PVA and Fe_3_O_4_. (e) The hysteresis loop of Fe_3_O_4_/PVA and (f) the zoom-in of the hysteresis loop of Fe_3_O_4_/PVA.

The morphology of the Fe_3_O_4_/PVA nanocomposite was investigated using SEM and TEM images, as shown in Fig. 1a and b, respectively. The nanoparticles exhibit a predominantly spherical shape with a uniform size distribution. Particle size analysis (Fig. 1c) indicates that the diameters range from 16 to 26 nm, with an average particle diameter calculated from the TEM image to be 20.054 nm, confirming the formation of nanoscale features essential for photothermal applications.

The crystallographic structure of the Fe_3_O_4_ core was confirmed by the XRD pattern, as shown in Fig. 1d. The diffraction pattern exhibits distinct peaks at 2*θ* values corresponding to the (220), (311), (400), (422), (511), and (440) planes, which are characteristic of the spinel crystal structure of magnetite (Fe_3_O_4_), consistent with JCPDS no. 19-0629.^30^ Upon incorporation of polyvinyl alcohol (PVA), an additional diffraction peak appears at 2*θ* ≈ 19.8°, which corresponds to the (101) plane of the semi-crystalline structure of PVA and confirms its successful deposition onto the surface of the Fe_3_O_4_ nanoparticles. The emergence of this peak provides direct evidence of the hybrid nature of the nanocomposite and supports the formation of a Fe_3_O_4_/PVA core–shell structure. This structural integration is essential for enhancing hydrophilicity, improving water transport, and enabling magnetic responsiveness in solar steam generation applications.

Fig. 1e and f present the magnetic properties of Fe_3_O_4_/PVA. The Fe_3_O_4_/PVA nanocomposite exhibits a saturation magnetization of 43 emu g^−1^ and a coercivity of 61 Oe, confirming the superparamagnetic behavior of the nanoparticles with soft magnetic characteristics. These magnetic properties are critical for enabling the field-responsive assembly of the nanocomposite into functional 3D structures during solar steam generation.

**Fig. 2:**
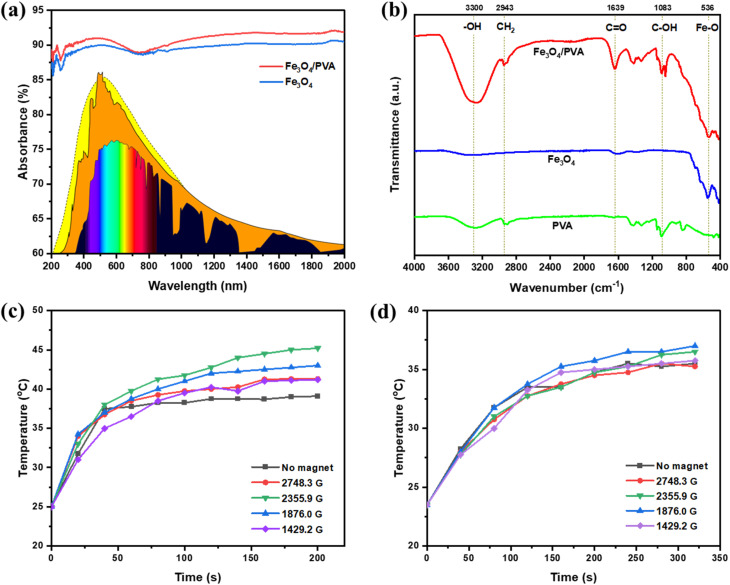
(a) UV-vis spectra of Fe_3_O_4_ and Fe_3_O_4_/PVA. (b) The FT-IR spectra of the synthesized materials. The increase of surface temperature of Fe_3_O_4_/PVA structure under different magnetic fields in the dry state (c) and in the wet state (d).

The optical absorption characteristics of the Fe_3_O_4_/PVA composite were assessed using UV-vis-NIR spectroscopy, and the results are shown in Fig. 2a. Pure Fe_3_O_4_ nanoparticles display strong broadband absorption, with approximately 85–90% of incident light absorbed across the full spectral range of 200–2000 nm. The increase in absorbance upon incorporating PVA into the Fe_3_O_4_ hybrid can be attributed to two primary factors. First, although PVA is generally transparent in the visible spectrum, it can interact with the nanoparticles to enhance overall light absorption through interfacial effects or minor contributions in the UV-near visible range, leading to the observed 2–3% broadband increase. Second, the PVA matrix promotes aggregation of Fe_3_O_4_ nanoparticles,^31^ which increases their effective size and reduces quantum confinement effects, thereby decreasing the bandgap and facilitating greater sunlight absorption across longer wavelengths.^31,32^ This makes it more efficient for the nanoparticles to harvest solar energy.

Notably, the incorporation of PVA into the composite results in a further enhancement of light absorption. The Fe_3_O_4_/PVA hybrid exhibits a 2–3% increase in absorbance across the entire wavelength range compared to bare Fe_3_O_4_. This enhancement can be attributed to the interaction between the Fe_3_O_4_ nanoparticles and the PVA polymer matrix. The improved absorption may arise from a slight increase in nanoparticle size caused by the encapsulating PVA network. This structural modification can lead to a reduction in the bandgap energy of Fe_3_O_4_, allowing more efficient photon absorption, particularly in the near-infrared region. These effects contribute synergistically to the superior light-harvesting capability of the Fe_3_O_4_/PVA composite, which is crucial for enhancing photothermal conversion efficiency in solar steam generation systems.

FTIR spectroscopy was conducted to examine the surface chemistry and confirm the successful functionalization of Fe_3_O_4_ nanoparticles with PVA. As shown in Fig. 2b, distinct spectral differences are observed between the unmodified Fe_3_O_4_ and the Fe_3_O_4_/PVA composite. The FTIR spectrum of bare Fe_3_O_4_ exhibits a characteristic split absorption band centered around 580 cm^−1^, which corresponds to the Fe–O stretching vibration.^33^ Upon modification with PVA, the composite spectrum reveals several new absorption bands associated with the polymer matrix. A broad and intense band appears around 3600 cm^−1^, attributable to the stretching vibration of hydroxyl (–OH) groups from the PVA backbone.^34^ Additionally, a distinct peak at 1639 cm^−1^ corresponds to the C=O stretching vibration, which is indicative of residual acetate groups from the PVA structure.^35^ The presence of a peak at 1083 cm^−1^, assigned to C–OH stretching vibrations,^35^ further supports the incorporation of PVA onto the Fe_3_O_4_ surface.

These findings confirm the successful surface functionalization of Fe_3_O_4_ nanoparticles with PVA. Importantly, the abundance of hydrophilic functional groups (–OH, C=O, and C–OH) on the composite surface facilitates efficient water adsorption and capillary transport. This hydrophilic character is crucial for maintaining a continuous water supply at the air–water interface during solar steam generation, thereby enhancing the evaporation rate and ensuring stable photothermal performance under solar illumination.^8^

Fig. 2c and d illustrate the changes in surface temperature for various membranes in the dry state (Fig. 2c) and wet state (Fig. 2d). It is evident that the application of magnetic fields leads to a significant increase in surface temperature in both states. Specifically, in the dry state, the maximum temperature reaches approximately 45 °C at a magnetic field strength of 2355.9 G, which is about 10 °C higher than that of the membrane without any magnetic field. Similarly, in the wet state, the peak surface temperature is around 37.5 °C under 1876 G. However, the temperature rise is not directly proportional to the increase in magnetic field strength. For instance, at a higher field of 2748.3 G, the surface temperature is lower than the values observed at 1876.0 G and 2355.9 G. This variation in surface temperature arises from the rearrangement of the membrane's surface structure induced by the magnetic field, which will be discussed in detail in the subsequent figures.

**Fig. 3:**
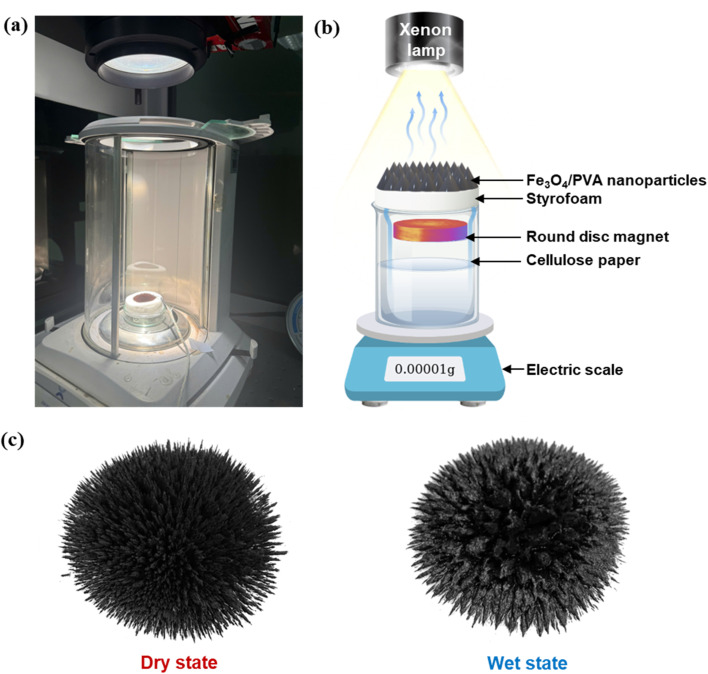
(a) The digital photo of the solar steam generation setup. (b) The solar steam generation setup. (c) The digital photos of Fe_3_O_4_/PVA spatial structures under 2355.9 G in dry and wet states.

Fig. 3a and b display the experimental setup used to evaluate the solar steam generation (SSG) performance of the Fe_3_O_4_/PVA membranes. A xenon lamp was employed as a solar simulator to provide consistent and controllable illumination equivalent to 1 sun. To induce the formation of three-dimensional microstructures, a round disc-shaped permanent magnet was positioned directly beneath a layer of styrofoam, which served as the floating support for the photothermal membrane.

The magnetic field generated by the disc magnet penetrated the styrofoam layer, initiating the self-assembly of Fe_3_O_4_ nanoparticles embedded within the PVA matrix into vertically oriented, needle-like structures. This magnetic response facilitated the spontaneous formation of a reconfigurable 3D surface architecture. During the evaporation process, water was continuously delivered from the bulk reservoir to the photothermal layer using a strip of cellulose paper, which functioned as a capillary-driven water supply channel to ensure stable and uninterrupted hydration. As shown in Fig. 3c, the Fe_3_O_4_/PVA membrane exposed to a magnetic field strength of 1876 G exhibits a well-defined needle-like 3D structure in both wet and dry states. This configuration enhances light absorption and interfacial heat localization, forming the basis for the membrane's high evaporation efficiency.

**Fig. 4:**
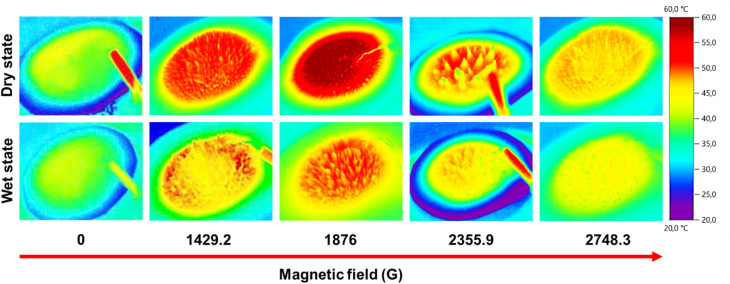
The photothermal images of different Fe_3_O_4_/PVA spatial structures in wet and dry states.

Fig. 4 presents the infrared thermal images of Fe_3_O_4_/PVA membranes subjected to varying magnetic field strengths, illustrating the strong correlation between magnetic field-induced structural changes and the photothermal performance of the material. The surface morphology and heat distribution evolve significantly as the magnetic field is modulated, directly impacting the efficiency of solar-to-thermal energy conversion. In the absence of an external magnetic field, the Fe_3_O_4_/PVA membrane remains in a flat, uniform configuration. This planar morphology exhibits limited light-trapping capability and heat localization, resulting in relatively low surface temperatures of approximately 40 °C in the wet state and 45 °C in the dry state. These values serve as a baseline for evaluating the enhancements achieved through magnetic manipulation.

Upon application of a moderate magnetic field of 1429.2 G, a pronounced morphological transformation occurs. The magnetic nanoparticles within the PVA matrix self-assemble into vertically oriented, needle-like microstructures, forming a three-dimensional (3D) architecture. This anisotropic configuration enhances multiple light scattering and internal reflection among the needle arrays, thereby improving solar absorption and reducing radiative and convective heat losses. As a result, the surface temperature increases notably, reaching approximately 50 °C in the wet state and 55 °C in the dry state. The formation of this 3D network represents an optimal balance between structural stability and thermal efficiency.

As the magnetic field strength increases further to 1876 G, the needle structures grow taller and more prominent. The enhanced geometric features improve localized heat concentration across a broader surface area, with a higher proportion of the membrane reaching temperatures above 55 °C (wet) and 60 °C (dry). At a higher magnetic field (2355.9 G), while the structures increase in height, their spatial density begins to decline due to excessive magnetic force pulling nanoparticles into elongated but loosely packed formations. This reduced density negatively affects the heat-retention characteristics, as evidenced by a slight drop in maximum surface temperature to around 40 °C in the wet state and a decrease in the area exhibiting temperatures in the 45–50 °C range.

At a field strength of 2748.3 G, the structural integrity of the needle-like architecture deteriorates significantly. The magnetic interactions become excessively strong, disrupting the internal cohesion of the assembled microstructures and leading to partial collapse or aggregation. The resulting morphology is less ordered and loses the favorable geometry for light trapping and heat localization. Consequently, the photothermal performance declines, with surface temperatures falling to approximately 45 °C (wet) and 50 °C (dry), which approaches the baseline condition observed without magnetic field assistance.

These results collectively demonstrate that the Fe_3_O_4_/PVA system exhibits strong magnetic field responsiveness, allowing precise control over microstructural assembly and thermal output. Moderate magnetic field strengths (around 1876 G) enable the formation of optimal 3D architectures that enhance light absorption and heat retention. In contrast, excessive field intensities compromise structural uniformity, reducing effective photothermal conversion. This magnetic tunability offers a promising strategy for engineering reconfigurable photothermal membranes tailored for high-efficiency solar steam generation.

**Fig. 5:**
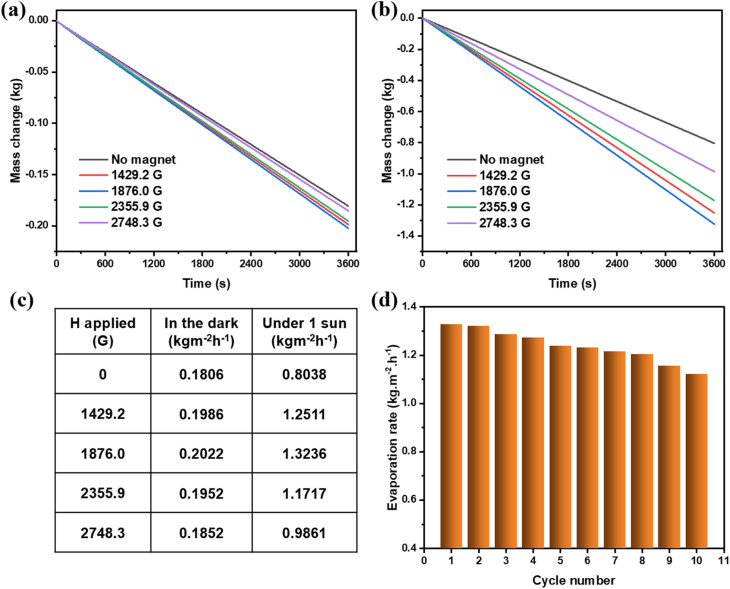
The evaporation rates of different Fe_3_O_4_/PVA structures (a) in the dark and (b) under 1 sun irradiation. (c) The evaporation rate of Fe_3_O_4_/PVA structures in the dark and under 1 sun irradiation. (d) The change in the evaporation rate after 10 cycles.

Fig. 5 presents the evaporation behavior of Fe_3_O_4_/PVA membranes subjected to varying magnetic field strengths, both in the absence of light (Fig. 5a) and under 1 sun illumination (Fig. 5b). In the dark, evaporation rates exhibit only minor variation with changing magnetic field intensity. As shown in Fig. 5c, the evaporation rate fluctuates slightly between 0.1806 and 0.2022 kg m^−2^ h^−1^ across field strengths from 0 to 2748.3 G. This narrow range suggests that, without photothermal activation, the magnetic field influences evaporation primarily through microstructural modifications that weakly affect capillary-driven water transport.

Under 1 sun irradiation, however, a markedly different trend is observed. The evaporation rate increases significantly with the introduction of a magnetic field, reaching a maximum of 1.3236 kg m^−2^ h^−1^ at 1876 G with a 64.67% enhancement compared to the baseline value of 0.8038 kg m^−2^ h^−1^ recorded without a magnetic field (0 G). The solar efficiency was calculated to be 93.30%. This dramatic increase confirms the critical role of magnetic field-induced 3D structuring in improving photothermal conversion efficiency and interfacial evaporation. Beyond the optimal field strength, the enhancement effect begins to diminish. At 2355.9 G and 2748.3 G, the evaporation rates decrease to 1.1717 and 0.9861 kg m^−2^ h^−1^, respectively, indicating that excessive magnetic fields may disrupt the structural integrity or reduce the density of the needle-like architecture, thereby compromising thermal localization and water supply pathways.

The long-term operational stability of the Fe_3_O_4_/PVA evaporator was also evaluated over 10 continuous evaporation cycles under 1 sun (Fig. 5d). A slight reduction in performance was observed, with the evaporation rate decreasing from 1.3236 to 1.1210 kg m^−2^ h^−1^ after 10 cycles. This modest decline reflects excellent structural durability and reusability of the magnetic nanohybrid system, demonstrating its potential for repeated use in practical solar water purification applications.

To elucidate the influence of a magnetic field on the structural behavior of a magnetic photothermal membrane, we calculated the magnetic force acting on a magnetic particle. In the case of a magnetic sphere placed within a non-uniform magnetic field, a force (*F*_p_) is generated due to the interaction between the particle's magnetic dipole moment and the spatial gradient of the magnetic field.^36^

1 *F*_p_ = *µ*_0_*V*_p_*M*_p_∇(*H*_p_)

where: *F*_p_ is the magnetic attraction force on a particle. *µ*_0_ is the permeability of vacuum. *V*_p_ is the particle volume. ∇(*H*_p_) is the gradient in the center of the gravity of the particle.

**Fig. 6:**
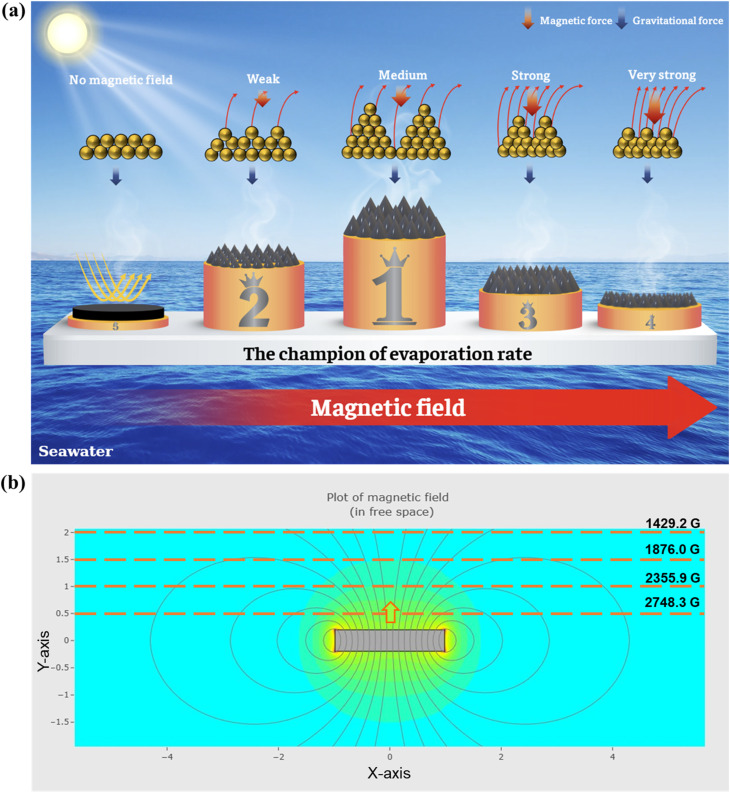
(a) The hypothesis explains the effect of a magnetic field on the structure of magnetic nanoparticles. (b) The simulated magnetic field distribution around a round disk magnet.

According to eqn (1), the magnetic force exerted on a particle increases with both the magnetic field strength and the spatial gradient of the field ∇(*H*_p_), particularly when the particle is located closer to the magnetic source. This is due to the stronger variation in magnetic flux density near the magnet surface. Furthermore, magnetic nanoparticles, such as Fe_3_O_4_, tend to align along the magnetic field lines due to dipole–dipole interactions and magnetic torque. As a result, their alignment and aggregation under the influence of the external field lead to the formation of chain-like or needle-like assemblies, which manifest as spike-like 3D microstructures, as observed in Fig. 6a.

Under a weak magnetic field, such as 1429.2 G (at a distance of 2 cm), the magnetic flux density is relatively low, and field lines are widely spaced (Fig. 6b). This results in a minimal magnetic gradient and consequently a weak magnetic force, insufficient to drive the formation of vertically aligned 3D structures. At an intermediate field strength, such as 1876 G, the magnetic flux becomes more concentrated, generating stronger magnetic dipole interactions and promoting the assembly of nanoparticles into vertically oriented, needle-like architectures aligned with the field direction.

When the applied field increases to 2355.9 G, the magnetic flux density and gradient further intensify, enhancing the magnetic attraction between particles and promoting the formation of a densely packed structure with longer and thicker needle-like features. However, the enlarged spacing between these structures increases the reflectivity and reduces the effective absorption of incident solar radiation, leading to decreased photothermal efficiency and a lower surface temperature.

At even higher field strengths, such as 2748.3 G (distance = 0.5 cm), the magnetic force becomes excessively strong, causing structural collapse due to gravitational compression or interparticle overload. The result is a significant reduction in the height and integrity of the needle-like features, which compromises the photothermal performance. Consequently, both the surface temperature and the water evaporation rate of the Fe_3_O_4_/PVA membrane decline substantially under such high magnetic field conditions.

## 4. Conclusion

In conclusion, the influence of external magnetic field strength on the structural configuration and photothermal performance of Fe_3_O_4_/PVA-based solar evaporators was systematically investigated. The results reveal that a moderate magnetic field of 1876 G effectively induces the formation of vertically aligned, needle-like 3D microstructures. This optimized architecture significantly enhances light absorption, promotes localized heat confinement, and increases the surface temperature, resulting in a peak water evaporation rate of 1.3236 kg m^−2^ h^−1^ under 1 sun illumination with the solar efficiency of 93.30%. In contrast, higher magnetic field strengths (≥2355.9 G) lead to structural distortion and decreased morphological stability, which compromise thermal localization and reduce overall evaporation efficiency. These observations underscore the critical role of magnetic field tuning in modulating the self-assembly and performance of magnetically responsive photothermal membranes. The insights gained here offer valuable design guidelines for the development of high-efficiency, reconfigurable solar steam generation systems suitable for scalable and sustainable water purification technologies.

## Conflicts of interest

The authors declare that they have no known competing financial interests or personal relationships that could have appeared to influence the work reported in this article.

## Data Availability

Data available on request from the authors.
